# Mechanical Fault Diagnosis of HVCBs Based on Multi-Feature Entropy Fusion and Hybrid Classifier

**DOI:** 10.3390/e20110847

**Published:** 2018-11-05

**Authors:** Shuting Wan, Lei Chen, Longjiang Dou, Jianping Zhou

**Affiliations:** 1 Department of Mechanical Engineering, North China Electric Power University, Baoding 071003, China; 2 Maintenance Company of State Grid Zhejiang Electric Power Company, Hangzhou 310000, China

**Keywords:** high-voltage circuit breaker, mechanical fault diagnosis, multi-feature entropy fusion, variational mode decomposition, principle components analysis, support vector data description

## Abstract

As high-voltage circuit breakers (HVCBs) are directly related to the safety and the stability of a power grid, it is of great significance to carry out fault diagnoses of HVCBs. To accurately identify operating states of HVCBs, a novel mechanical fault diagnosis method of HVCBs based on multi-feature entropy fusion (MFEF) and a hybrid classifier is proposed. MFEF involves the decomposition of vibration signals of HVCBs into several intrinsic mode functions using variational mode decomposition (VMD) and the calculation of multi-feature entropy by the integration of three Shannon entropies. Principle component analysis (PCA) is then used to reduce the dimension of the multi-feature entropy to achieve an effective fusion of features for selecting the feature vector. The detection of an unknown fault in HVCBs is achieved using support vector data description (SVDD) trained by normal-state samples and specific fault samples. On this basis, the identification and classification of the known states are realized by the support vector machine (SVM). Three faults (i.e., closing spring force decrease fault, buffer spring invalid fault, opening spring force decrease fault) are simulated on a real SF6 HVCB to test the feasibility of the proposed method. The detection accuracies of the unknown fault are 100%, 87.5%, and 100% respectively when each of the three faults is assumed to be the unknown fault. The comparative experiments show that SVM has no ability to detect the unknown fault, and that one-class support vector machine (OCSVM) has a weaker ability to detect the unknown fault than SVDD. For known-state classification, the adoption of the MFEF method achieved an accuracy of 100%, while the use of a single-feature method only achieved an accuracy of 75%. These results indicate that the proposed method combining MFEF with hybrid classifier is thus more efficient and robust than traditional methods.

## 1. Introduction

As an essential protection link in a power system, the operation states of high-voltage circuit breakers (HVCBs) are directly related to the stability and the safety of the power system. HVCB faults may not only cause huge economic losses, but also have negative social impact [1,2]. Thus, maintenance for HVCBs has become a daily task. However, traditional scheduled maintenance is time-consuming and may not ensure the reliability of HVCBs when they are over-maintained [3,4]. It is thus necessary to adopt more simple methods for performing fault diagnoses of HVCBs.

Most of the reported faults of HVCBs are mechanical in nature [5]. Recent studies on HVCB fault diagnosis mainly focus three aspects: vibration signals analysis [6,7,8,9,10], electromagnet coil current analysis [11,12], and dynamic simulation analysis [13,14]. Due to the operating characteristics of HVCBs, the accurate electromagnet coil currents are difficult collect, and the accurate size of mechanical structures for dynamic simulation analysis are difficult to obtain, which limits the applications of these two aspects in HVCB fault diagnoses. On the other hand, as a non-intrusive fault diagnosis method, vibration signal analysis has seen many applications in HVCB fault diagnoses. Runde et al. [15] analyzed the feasibility of using vibration signals for fault diagnoses of HVCBs. Subsequent studies usually map high-dimensional vibration signals to low-dimensional feature vectors by signal processing, which are then fed into the classifier to identify the fault type [16]. A vibration signal displays high complexity where the fault information may exist in different frequency components. It is thus necessary to perform a multi-scale decomposition on vibration signals using various methods, including wavelet packet decomposition (WPD), empirical mode decomposition (EMD), and local mean decomposition (LMD). Ma et al. [17] proposed a fault diagnosis method based on WPD and a random forest classifier. This method achieved classification accuracy of up to 95.56%. Liu et al. [18] applied EMD-entropy to extract feature vectors from vibration signals of a HVCB. Huang et al. [19] constructed a hybrid method based on LMD and time segmentation energy entropy. Although the above methods have achieved good results, there are still some inadequacies. WPD has heavy requirements for the selection of wavelet basis function, while the EMD and the LMD methods are sensitive to noise and sampling.

Variational mode decomposition (VMD) is a new adaptive signal processing method [20]. VMD has a solid mathematical foundation and it can adaptively decompose a signal to a specified number of intrinsic mode functions (IMFs). Each IMF and its corresponding center frequency are updated by constructing and solving variational problems. VMD has been successfully applied in the field of fault diagnosis.

After signals are decomposed, features are extracted to prepare for subsequent recognition and classification of the faults. Most current studies focus on utilizing a single feature for fault diagnosis. The single features include energy entropy, singular spectrum entropy, and sample entropy. Low accuracy and poor robustness are known problems of methods utilizing the single feature [21,22]. Thus, to resolve issues associated with methods based on a single feature, the multi-feature entropy fusion (MFEF) vectors of vibration signals by the integration of three Shannon entropies are proposed in this paper for the extraction of feature vectors. The feature vectors can reflect the characteristics of signals from the perspective of energy characteristic, mutation, and complexity. 

For the methods of fault recognition, neural networks (NNs) [23] and support vector machine (SVM) [24,25,26] are commonly used. Although NNs have good anti-noise and self-learning ability, they require a large number of samples to train, while HVCBs cannot operate frequently due to their working characteristics. SVM constructs an optimal hyperplane in feature space based on structural risk minimization theory [27,28]. Since SVM can effectively solve the problems of small samples, high dimension, and non-linearity, it is used to classify the mechanical faults in this paper. 

Previous research has contributed a lot to improving the classification accuracy of fault diagnosis when the fault is known. However, few studies have been made on the detection of unknown faults. In fact, it is impossible to record all HVCB faults to train the classifier. An unknown fault cannot be detected successfully by SVM because of the lack of training samples. Thus, it is necessary to detect whether an unknown fault occurs in HVCBs before fault classification. Support vector data description (SVDD) is a new, one-class classifier proposed by Tax [29], which is inspired by SVM. SVDD constructs an optimal hypersphere to classify the samples. If the feature vectors of samples are in the optimal hypersphere, they are regarded as known states. Otherwise, they are regarded as unknown faults.

This paper proposed a new method for fault diagnosis of HVCBs based on VMD-MFEF and a hybrid classifier. VMD is employed to decompose vibration signals into a specified number of IMFs. Then, MFEF vectors are calculated as the feature vectors. The hybrid classifier is constructed with SVDD and SVM. SVDD is trained by all normal state samples and all available fault samples to detect whether unknown faults occur in HVCBs. On this basis, the identification and classification of known faults are realized by SVM.

The paper is organized as follows. Section 2 introduces the mathematical model of VMD. Section 3 presents the extraction method of feature vectors. Section 4 introduces principles of SVM and SVDD. Section 5 illustrates the experimental application. Section 6 concludes on the proposed diagnosis method and includes some future directions.

## 2. Variational Mode Decomposition 

By solving the constrained variational problem, VMD can decompose a multi-component signal into a number of band-limited IMFs. Assuming that the multi-component signal *x*(*t*) is decomposed into *K* IMFs, the variational modal problem is constructed as follows:

(1) Hilbert transform is performed on each IMF to get its analytical signal.
  (1) $$\left(\delta (t)+\frac{j}{\pi t}\right)*u_{k}(t)$$
(2) Estimate the center frequency *ω_k_* of each IMF and mix them by frequency shifting, which can transform the frequency spectrum of each IMF to the baseband.
  (2) $$\left[\left(\delta (t)+\frac{j}{\pi t}\right)*u_{k}(t)\right]e^{-j\omega_{k}t}$$
(3) The bandwidth of each IMF is estimated through the squared *L*^2^-norm of a gradient. Consequently, the construction of the constrained variational problem can be described by Equation (3).
  (3) $$\left\{ \begin{array}{l} \underset{\left\{u_{k}\},\{w_{k}\right\}}{\min}\{{\sum_{k}\left\|\partial_{t}\left[\left(\delta (t)+\frac{j}{\pi t}\right)*u_{k}(t)\right]e^{-j\omega_{k}t}\right\|}^{2}\}\\ s.t\sum_{k}u_{k}=f \end{array}\right.$$
  where $\left\{u_{k}\}=\{u_{1},\cdots ,u_{K}\right\}$ and $\left\{\omega_{k}\}=\{\omega_{1},\cdots ,\omega_{K}\right\}$ are shorthand notations for the set of all IMFs and their center frequencies.
(4) Due to the difficulty of solving the constrained problem, the penalty parameter α and the Lagrange multiplier λ(t) are introduced to transform Equation (3) into an unconstrained variational problem, thereby obtaining an augmented Lagrange expression:
  (4) $$L({\{u}_{k}\},\{\omega_{k}\},\lambda )=\alpha {\sum_{k}\left\|\partial_{t}\left[\left(\delta (t)+\frac{j}{\pi t}\right)-u_{k}(t)\right]e^{-jw_{k}t}\right\|}_{2}^{2}+{\left\|f(t)-\sum_{k}u_{k}(t)\right\|}_{2}^{2}+\langle \lambda (t),f(t)-\sum_{k}u_{k}(t)\rangle$$

The above variational problem can be solved by alternating the direction method of multipliers, and the final solution of Equation (3) is obtained by alternately updating $u_{k}^{n+1}$, $\omega_{k}^{n+1}$, $\lambda_{k}^{n+1}$ and searching for the saddle point of Lagrange. Correspondingly, the IMFs *u_k_* and the center frequencies *ω_k_* are updated by Equations (5) and (6).

(5) $${\hat{u}}_{k}^{n+1}(\omega )=\frac{\hat{f}(\omega )-\sum_{i\neq k}{\hat{u}}_{i}(\omega )+\frac{\hat{\lambda }(\omega )}{2}}{1+2\alpha {\left(\omega -\omega_{k}\right)}^{2}}$$

(6) $$\omega_{k}^{n+1}=\frac{\int_{0}^{\infty }\omega {\left|{\hat{u}}_{k}(\omega )\right|}^{2}d\omega }{\int_{0}^{\infty }{\left|{\hat{u}}_{k}(\omega )\right|}^{2}d\omega }$$

The concrete implementation process of the VMD algorithm:

Step1: Initialize $\left\{{\hat{u}}_{k}^{1}\right\}$, $\left\{\omega_{k}^{1}\right\}$, $\left\{\lambda_{k}^{1}\right\}$, *n* = 0.

Step2: Update $u_{k}$ and $\omega_{k}$ according to Equations (5) and (6).

Step3: Update $\hat{\lambda }$ according to Equation (7).

(7) $${\hat{\lambda }}^{n+1}(\omega )\leftarrow {\hat{\lambda }}^{n}(\omega )+\tau [\hat{f}(\omega )-\sum_{k}{\hat{u}}_{k}^{n+1}(\omega )]$$

Step4: Repeat the iterative process of step 2 until convergence, namely:

(8) $$\sum_{k}\frac{{\left\|{\hat{u}}_{k}^{n+1}-{\hat{u}}_{k}^{n}\right\|}_{2}^{2}}{{\left\|{\hat{u}}_{k}^{n}\right\|}_{2}^{2}}<\varepsilon$$

## 3. Feature Extraction 

### 3.1. Multi-Feature Entropy

By using VMD, a multi-component signal can be decomposed into a set of IMFs. Considering that the vibration signals of HVCBs are nonlinear, non-stationary, and non-periodic, this paper adopted Shannon entropy to reflect the characteristics of vibration signals. Shannon entropy can effectively describe the disorder degree of a complicated nonlinear signal [30]. To ensure the accuracy and robustness of subsequent fault classification, multi-feature entropy is extracted based on three aspects: energy characteristic, mutation, and complexity. Then, three Shannon entropies are calculated to form a multi-feature entropy vector.

The envelope of a signal often contains information of sudden changes. Hilbert transform is used to extract the envelope of a signal. Suppose that the analytic signal of *x*(*t*) is defined as follows:

(9) g(t)=x(t)+jH[x(t)]

where H[x(t)] is the Hilbert transform form of *x*(*t*), and the amplitude of *g*(*t*) is the envelope of the signal *x*(*t*), the envelope values are obtained by Equation (10).

(10) $$A(t)=\sqrt{x^{2}(t)+H^{2}\left[x(t)\right]}$$

#### 3.1.1. Envelope Energy Entropy

The energy of vibration signals will change when different faults occur in HVCBs [8]. In this paper, envelope energy entropy (EEE) is adopted to calculate the energy entropy of HVCBs. Each IMF is divided into *M* segments on the time axis, and the envelope energy of each segment is calculated as Equation (11).

(11) $$Q(i)={\int_{t_{i}}^{t_{i}+T/M}\left|A(t)\right|}^{2}dt$$

where *i* = 1, 2, …, *M*; *t_i_* and *t_i_* + *T*/*M* represent the beginning time and the ending time of the *i*th segment signal. EEE of each IMF can be drawn according to the basic theory of Shannon entropy.

(12) $$H_{EE}=-\sum_{i=1}^{M}q(i)\ln q(i)$$

where, *q*(*i*) is the normalized energy value of the IMF and $\sum_{i=1}^{M}q_{i}=1$, which can be calculated by Equation (13).

(13) $$q(i)=\frac{Q(i)}{\sum_{i=1}^{M}Q(i)}$$

where $\sum_{i=1}^{M}Q(i)$ is the total energy of the IMF.

#### 3.1.2. Envelope Spectrum Entropy

The envelope spectrum can be obtained by performing a Fourier transform on an envelope signal. Envelope spectrum analysis can effectively reflect characteristics in the frequency domain of signals. The intensity of a signal envelope spectrum varies greatly under different conditions. Therefore, envelope spectrum entropy (ESE) is adopted to reflect the mutation of the vibration signals. ESE is calculated as follows.

(14) $$H_{ES}=-\sum_{i=1}^{M}p(i)\ln p(i)$$

where, *p*(*i*) is the normalized envelope spectrum value in the IMF and $\sum_{i=1}^{M}p_{i}=1$, which can be calculated by Equation (15).

(15) $$p(i)=\frac{HX(i)}{\sum_{i=1}^{N}HX(i)}$$

where, *HX*(*i*) is the envelope spectrum of the IMF, *I* = 1, 2, …, *N*, *N* is the sampling point for the signal.

#### 3.1.3. Multi-Resolution Singular Spectrum Entropy

Singular values can reflect the inherent characteristics of signals and have good stability. In this paper, multi-resolution singular spectrum entropy is used to mine the essence of vibration signals.

Firstly, let the reconstruction signal of IMF *k* be $D_{k}=\left\{u_{k}(j)\right\}$, from which *u_j_*(1), *u_j_*(2), ..., *u_j_*(*n*) is supposed to be the first vector of *n*-dimensional phase space. Then, take *u_j_*(2), *u_j_*(3), ..., *u_j_*(*n* + 1) as the second vector. By this analogy, an (*N* − *n* + 1) × *n* dimensional matrix G is constructed [31].

(16) $$G=\left[\begin{array}{cccc} u_{j}(1) & u_{j}(2) & \cdots & u_{j}(n)\\ u_{j}(2) & u_{j}(3) & \cdots & u_{j}(n+1)\\ \vdots & \vdots & \vdots & \vdots \\ u_{j}(N-n+1) & u_{j}(N-n+1) & \cdots & u_{j}(N) \end{array}\right]$$

Singular value decomposition (SVD) is conducted to decompose the matrix *G*. The decomposition result of the matrix $G_{\left(N-n+1\right)\times n}$ is $G=U_{(N-n+1)\times l}S_{l\times l}V_{l\times l}^{T}$. The nonzero diagonal elements *λ_i_* (*i* = 1, 2, …, *l*; *l* = min((*N* − *n* + 1), *n*)) from $S_{l\times l}$ are singular values of the matrix *G*. According to the theory of the Shannon entropy, multi-resolution singular spectrum entropy (MSSE) is calculated by Equation (17).

(17) $$H_{MSS}(k)=-\sum_{i=1}^{l}s(i)\ln s(i)$$

where, *s*(*i*) is the normalized singular value of the IMF and $\sum_{i=1}^{M}s_{i}=1$, which can be calculated by Equation (18).

(18) $$s(i)=\frac{\lambda (i)}{\sum_{i=1}^{l}\lambda (i)}$$

where, *λ*(*i*) is singular value of the IMF.

### 3.2. Principle Component Analysis

A multi-feature entropy vector can be formed by the integration of EEE, ESE, and MSSE. Due to the fact that there are many features in the feature space, the subsequent identification of faults will be affected by the information redundancy. PCA is used to optimize feature space to achieve effective fusion of the features.

PCA is a data dimensionality reduction method, whose basic idea is to reduce the dimension of the correlative index $\left[H_{1},H_{2},\cdots ,H_{s}\right]$ to a few unrelated, comprehensive indexes [32]. The comprehensive index should reflect the information represented by the original index to the greatest extent.

Suppose that $\left[F_{1},F_{2},\cdots ,F_{s}\right]$ represents *m* principal components (PCs) of the original variables $\left[H_{1},H_{2},\cdots ,H_{s}\right]$, then:

(19) $$\left\{ \begin{array}{l} F_{1}=a_{11}H_{1}+a_{12}H_{2}+\cdots +a_{1s}H_{s}\\ F_{2}=a_{21}H_{1}+a_{22}H_{2}+\cdots +a_{2s}H_{s}\\ \cdots \\ F_{m}=a_{m1}H_{1}+a_{m2}H_{2}+\cdots +a_{ms}H_{s} \end{array}\right.$$

## 4. Hybrid Classifier

In the actual operation environment, there may be some unrecorded unknown faults occurring in HVCBs. Once this happens, the classifier may recognize an unknown fault as the known state due to the lack of training samples of the unknown faults. Therefore, a hybrid classifier constructed with SVDD, and SVM is used for the faults diagnosis of HVCBs.

### 4.1. Principles of SVM

**Figure 1:**
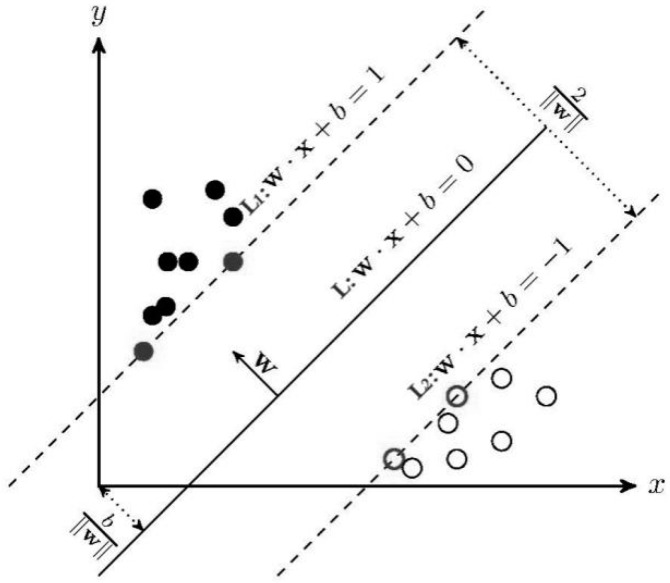
Classification of two classes using SVM.

SVM is a general machine-learning algorithm based on the principle of minimizing structural risk, which is suitable for the classification of small sample data. The basic idea of SVM is shown in Figure 1.

In Figure 1, the solid points and hollow points represent two types of training samples. SVM can correctly classify the two types of samples by constructing an optimal hyperplane and the classification interval of this optimal hyperplane is the largest. In Figure 1, line L is the optimal hyperplane, line L_1_ and line L_2_ are parallel to the optimal hyperplane L. The distance between L_1_ and L_2_ is the classification interval. The samples that satisfy L_1_ and L_2_ are called support vectors. Therefore, the problem of constructing the optimal hyperplane L can be transformed into the following optimization problem:

(20) $$\{\begin{array}{c} \underset{w,b}{\min}\frac{1}{2}{\left\|w\right\|}^{2}\\ s.t.\begin{array}{cc} & y_{i}\left(w\cdot x+b\right)\geq 1,i=1,2,\ldots ,\;l \end{array} \end{array}$$

where *w* is the normal vectors of the optimal hyperplane, *b* is the threshold.

Generalized to linear indivisible cases, the slack variables $\xi_{i}$ and the penalty factor *C* are introduced to solve the problem that some samples cannot be classified correctly by the hyperplane. The generalized function is shown as:

(21) $$\{\begin{array}{c} \underset{w,b}{\min}\frac{1}{2}{\left\|w\right\|}^{2}+C\sum_{i=1}^{l}\xi_{i}\\ s.t.\begin{array}{cc} & y_{i}\left(w\cdot x+b\right)\geq 1-\xi_{i},i=1,2,\ldots ,\;l \end{array} \end{array}$$

The Lagrange multipliers are introduced to solve the above problems.

(22) $$\max L=\sum_{i=1}^{l}\alpha_{i}\alpha_{j}y_{i}y_{j}x_{i}^{T}x_{j}$$

The constraint of Equation (22) is as folllows.

(23) $$\sum_{i=1}^{l}\alpha_{i}y_{i}=0,0\leq \alpha_{i}\leq C$$

where $\alpha_{i}$ is Lagrange multiplier. For linear indivisible problems, low dimensional samples can be mapped to a higher dimension space by $K\left(x_{i},x_{i}\right)$ in Equation (24). The final optimal function is shown below:

(24) $$\max L=\sum_{i=1}^{l}\alpha_{i}-\frac{1}{2}\sum_{i=1,j=1}^{l}\alpha_{i}\alpha_{j}y_{i}y_{j}K\left(x_{i},x_{j}\right)$$

### 4.2. Principles of SVDD

SVDD is a new one-class classifier which is inspired by SVM. SVDD is put forward to detect novel data or outliers. The main idea of SVDD is to construct a hypersphere with the minimum volume that can contain all the samples as much as possible.

Let $Q=\left\{x_{i}|i=1,\ldots ,n\right\}$ be the training vector, so the minimum volume hypersphere can be expressed as follows [33]:

(25) $$\begin{array}{l} \min f\left(a,R,\xi_{i}\right)=R^{2}+C\sum_{i}^{n}\xi_{i}\\ s.t.{\left(x_{i}-a\right)}^{T}\left(x_{i}-a\right)\leq R^{2}+\xi_{i},\xi_{i}\geq 0 \end{array}$$

where *R* is the radius of the hypersphere, *a* is the center of the hypersphere, $\xi_{i}$ is the slack variable, and *C* is the penalty factor which controls the balance between the volume of the hypersphere and the number of rejected points.

To solve the above convex quadratic optimization problem, the Lagrange equation is constructed as follows:

(26) $$f\left(a,R,\alpha_{i},\xi_{i}\right)=R^{2}+C\sum_{i}^{n}\xi_{i}-\sum_{i}\alpha_{i}\left[R^{2}+\xi_{i}^{2}-{\left(x_{i}-a\right)}^{2}\right]-\sum_{i}\gamma_{i}\xi_{i}$$

where $\alpha_{i}$ and $\gamma_{i}$ are the Lagrange multipliers, $\alpha_{i}\geq 0$, $\gamma_{i}\geq 0$.

Letting the partial derivatives of the Equation (26) with respect to the variables (*R*, *α, ξ_i_*) equal to 0, the dual form of the optimization problem can be thus solved as follows:

(27) $$\begin{array}{l} \min f=\sum_{i}\alpha_{i}K\left(x_{i},x_{i}\right)-\sum_{i,j}^{n}\alpha_{i}\alpha_{j}K\left(x_{i},x_{j}\right)\\ s.t.\;0\leq \alpha_{i}\leq C,\sum_{i=1}^{n}\alpha_{i} \end{array}$$

where *K*(*x_i_*,*x_i_*) is RBF kernel function. A set of Lagrange multiplier *α_i_* can be obtained by solving Equation (27), and samples *x_i_* are called support vectors with *α_i_* > 0. The square distance *D* from a sample to the center of the hypersphere is calculated bellow:

(28) $$D=K\left(x,x\right)-2\sum_{i=1}^{n}\alpha_{i}K\left(x_{i},x\right)+\sum_{i=1}^{n}\sum_{j=1}^{n}\alpha_{i}\alpha_{j}K\left(x_{i},x_{j}\right)$$

Equation (29) is defined to judge whether the test samples are in the hypersphere:

(29) $$f_{\mathrm{svdd}}=\mathrm{sgn}\left(R^{2}-{\left\|x_{i}-a\right\|}^{2}\right)=\mathrm{sgn}\left(R^{2}-D\right)$$

### 4.3. Fault Diagnosis Process

This paper proposed a new method for fault diagnosis of HVCBs based on VMD-MFEF and a hybrid classifier. VMD is employed to decompose vibration signals into a specified number of IMFs. Three entropies are calculated to form the multi-feature vector. PCA is used to reduce the dimension of the multi-feature vectors. The hybrid classifier is constructed with SVDD and SVM which are trained by all normal state samples and all available fault samples. The fault diagnosis steps are described below.

(1) Decompose the vibration signal into *K* IMFs by VMD.
(2) Calculate EEE, ESE, and MSSE of signals according to Equations (11)–(18) to form the multi-feature entropy vector.
(3) Use PCA to reduce the dimension of the multi-feature entropy vectors.
(4) Use SVDD to diagnosis whether unknown faults occur in HVCBs by solving Equation (29). If *f*(*x*) > 0, the sample is imported into the area of known faults; otherwise, it is imported into the area of unknown faults.
(5) Use SVM to classify the fault type in known states area.

**Figure 2:**
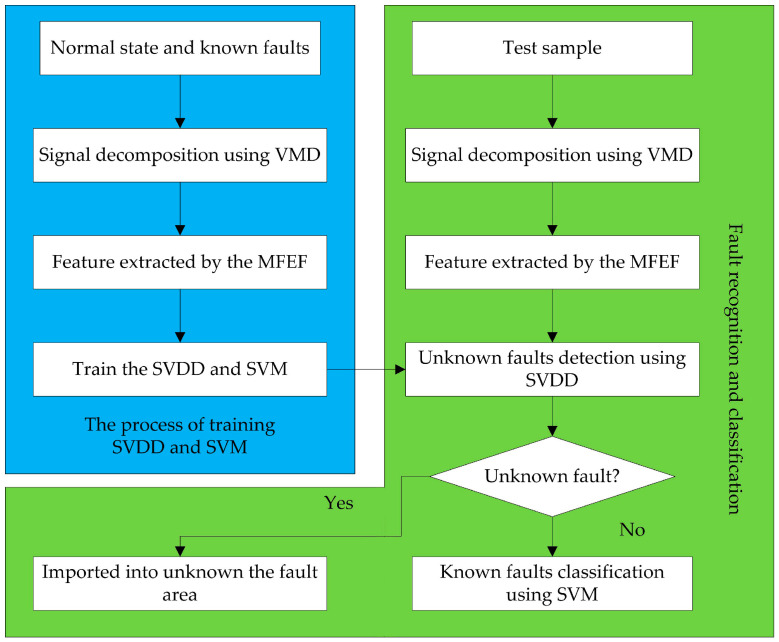
Fault diagnosis process of the proposed method.

Figure 2 shows the process of the diagnosis method.

## 5. Experimental Application

### 5.1. Data Acquisition

**Figure 3:**
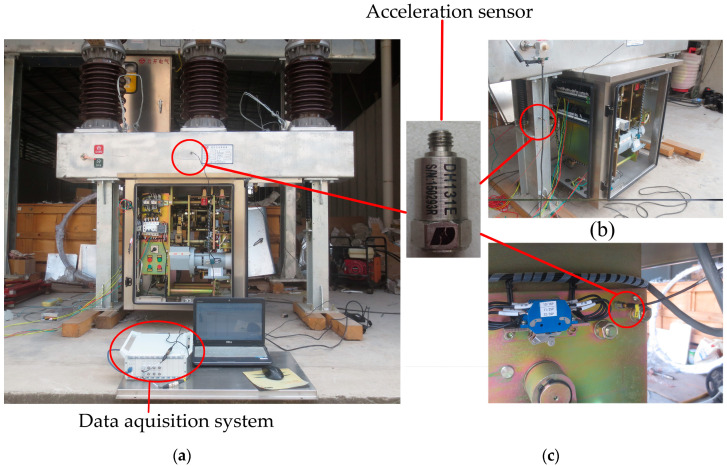
Positions for installing the acceleration sensor. (**a**) The beam near the operating box; (**b**) The base; (**c**) The operating box.

The experiment is conducted on an outdoor high-voltage SF_6_ circuit breaker, as shown in Figure 3. The DH131E piezoelectric acceleration sensor produced by Donghua Testing Technology Company is used for the vibration signal acquisition of the HVCB. The acceleration sensor is monoaxial. The specific parameters of the acceleration sensor are as follows: frequency response: 1–8000 Hz; measure range: 500 g. The sensor installation position must meet the following three principles: (1) the sensor does not affect the normal operation of the HVCB; (2) the sensor position is close to the structures which are most concerned; (3) the sensor on the selected position can collect signals stably and repeatedly. In order to find a good installation position for the sensor, three different positions of the HVCB are installed with sensors to collect vibration signals for comparison. The three positions are the beam, the base, and the operation box, as shown in Figure 3.

**Figure 4:**
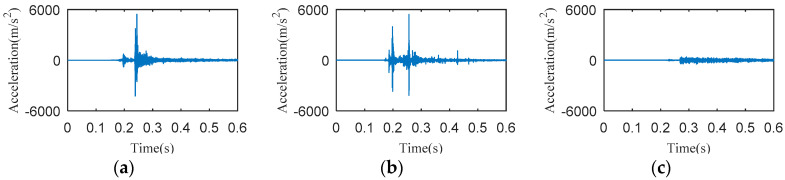
Vibration signals at three different positions. (**a**) The beam near the operating box; (**b**) The operating box; (**c**) The base.

The data acquisition card is used to record the data a 10 kHz sampling frequency for a time period of 600 ms during the closing operation. Typical vibration signals collected at these three places are shown in Figure 4.

From Figure 4, it can be seen that the intensity of the vibration signal collected at the base is too small to be used for analysis. Moreover, the vibration signal starting time of the base lags behind that of the operation box and the beam. This indicates that the transmission distance from the vibration source to the base is so long that the vibration energy is greatly attenuated. In fact, the vibration signals collected at the beam and the operating box are all available for subsequent analysis. Considering the convenience and the repeatability of signal collections, the beam is finally selected as the installation position of the acceleration sensor. In addition, there is an opening spring on each side of the HVCB; when the sensor is installed on one side, the vibration signals transmitted from the other side will be very weak, which is not conducive to analysis. Considering the above factors, the acceleration sensor is installed on the middle of the beam near the operating box, as shown in Figure 3a. The selected acceleration sensor installation position is close to the buffer spring and the closing boring. And there is an almost equal distance from the acceleration sensor to the opening springs of the two sides.

**Figure 5:**
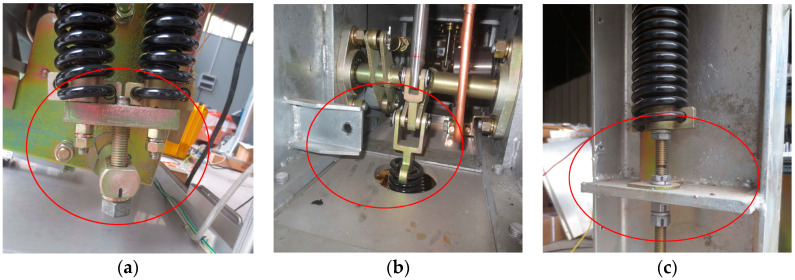
Simulative experiments of fault patterns. (**a**) Fault I; (**b**) Fault II; (**c**) Fault III.

In order to verify the effectiveness of the fault diagnosis method proposed in this paper, three spring faults of the HVCB are simulated in field experiments: (1) closing spring force decrease fault (Fault I); (2) buffer spring invalid fault (Fault II); (3) opening spring force decrease fault (Fault III). Meanwhile, the vibration signals under normal state are collected. Fault I is simulated by adjusting the closing spring tension, as shown in Figure 5a. Fault II is simulated by removing the buffer spring, as shown in Figure 5b. Fault III is simulated by adjusting the opening spring tension, as shown in Figure 5c.

**Figure 6:**
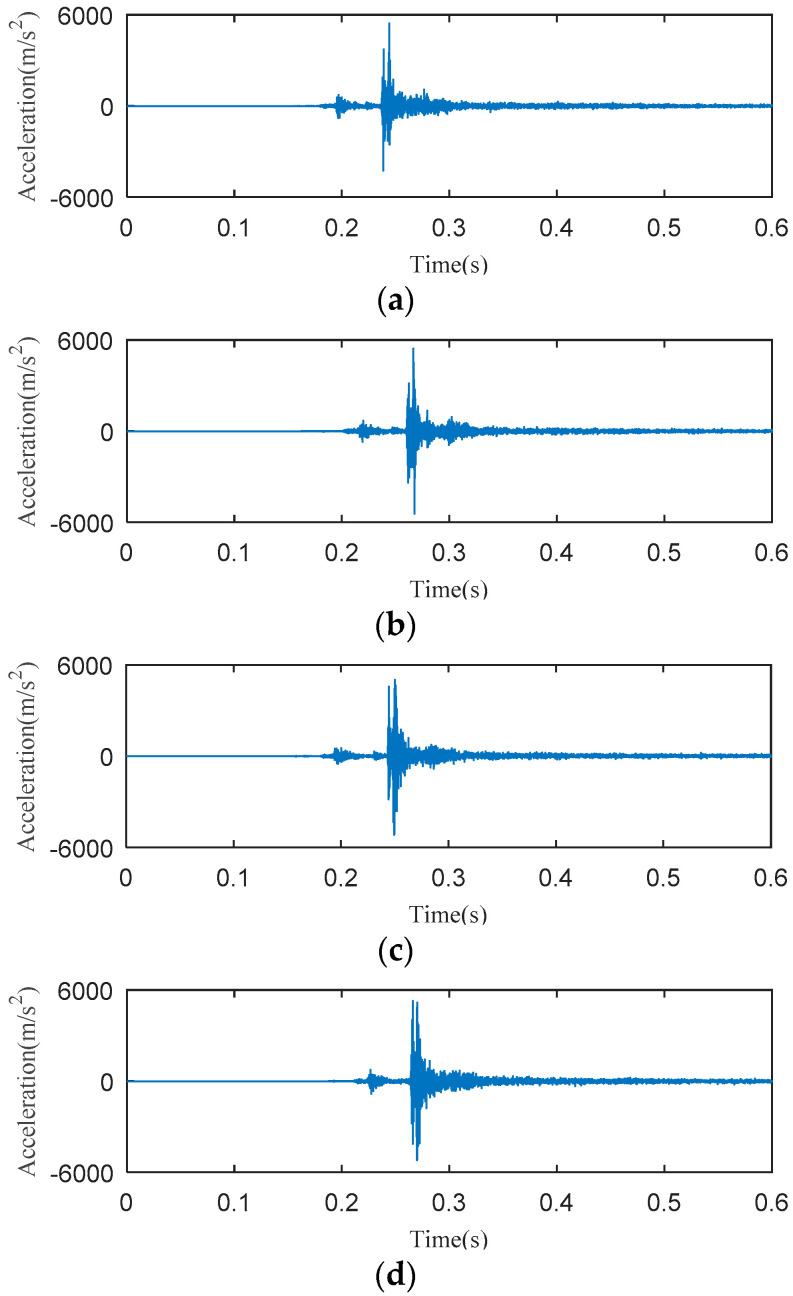
Vibration signals of the HVCB. (**a**) Normal state; (**b**) Fault I; (**c**) Fault II; (**d**) Fault III.

In order to avoid the HVCB damaged from excessive operations, 30 experiments of the normal state and 30 experiments per fault type are carried out to collect vibration signals. The typical vibration signal waveforms of four different mechanical states are shown in Figure 6.

As shown in Figure 6, the starting time of Faults I and III lags behind the normal state signal. The maximum amplitude of the normal state signal is slightly less than that of three faults. Despite these characteristics, it is difficult to correctly distinguish the mechanical state of the HVCB. Therefore, it is necessary to process the vibration signal to judge the mechanical state of the HVCB.

### 5.2. Signal Processing

**Figure 7:**
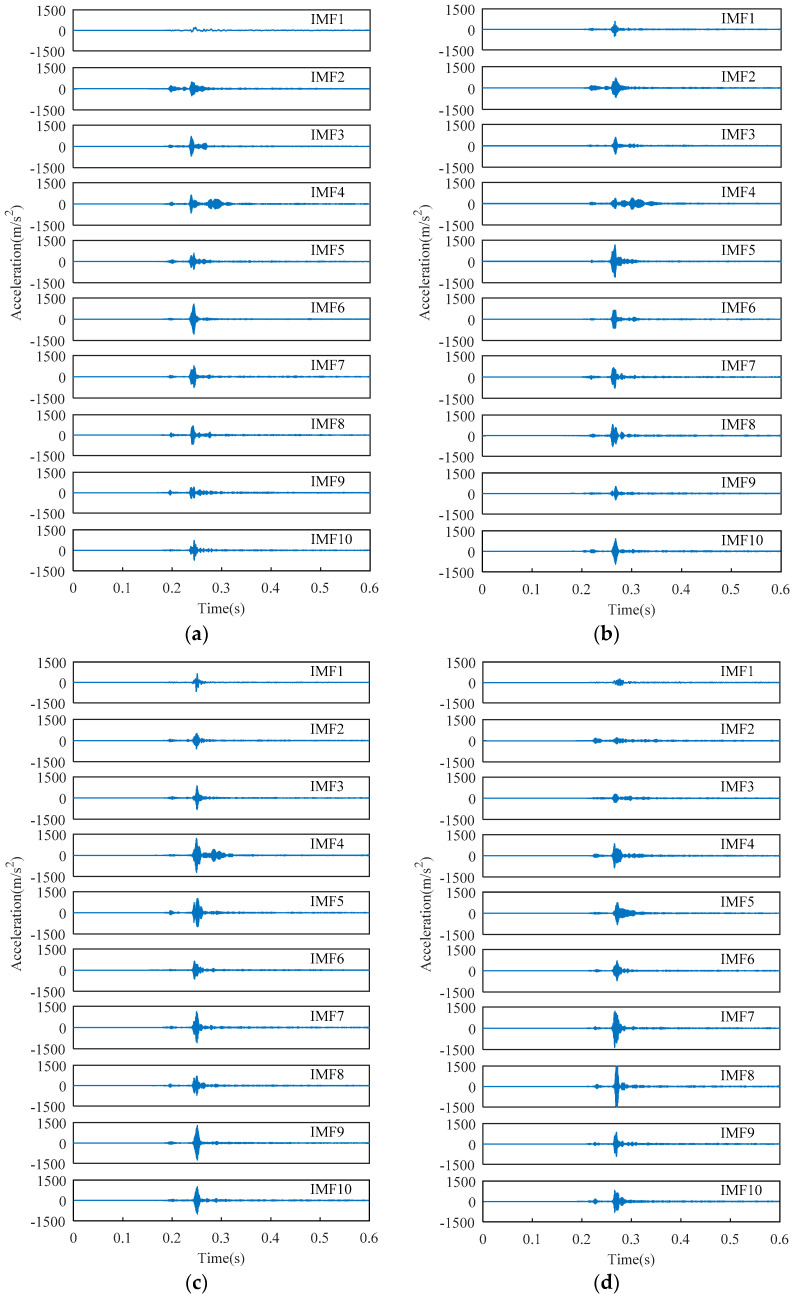
IMFs of the four types of vibration signals obtained by VMD. (**a**) Normal state; (**b**) Fault I; (**c**) Fault II; (**d**) Fault III.

VMD is employed to decompose vibration signals. The superiority of VMD has been demonstrated by Huang [16], and will not be repeated here. The decomposition layer of VMD is set to 10 layers since the center frequency of the eleventh IMF begins to be approximate to the center frequency of the tenth layer. The decomposition results of the vibration signals are shown in Figure 7.

Figure 7 indicates some characteristics of signals in the time domain or frequency domain. The amplitude of the last three IMFs of the normal state is less than that of the three faults. The fourth IMF of the normal state, Fault I, and Fault II has multiple energy centers.

### 5.3. Feature Extraction

#### 5.3.1. Multi-Feature Entropy Extraction

Multi-feature entropy can be extracted based on the three Shannon entropy methods (EEE, ESE, MSSE). Firstly, in order to calculate EEE, the IMF is divided into 10 segments on the time axis, and EEE of the IMF is calculated according to Equations (9)–(13). Secondly, ESE is calculated according to Equations (14) and (15). Thirdly, MSSE is calculated according to Equations (16)–(18) based on 100-dimensional phase space reconstruction.

**Figure 8:**
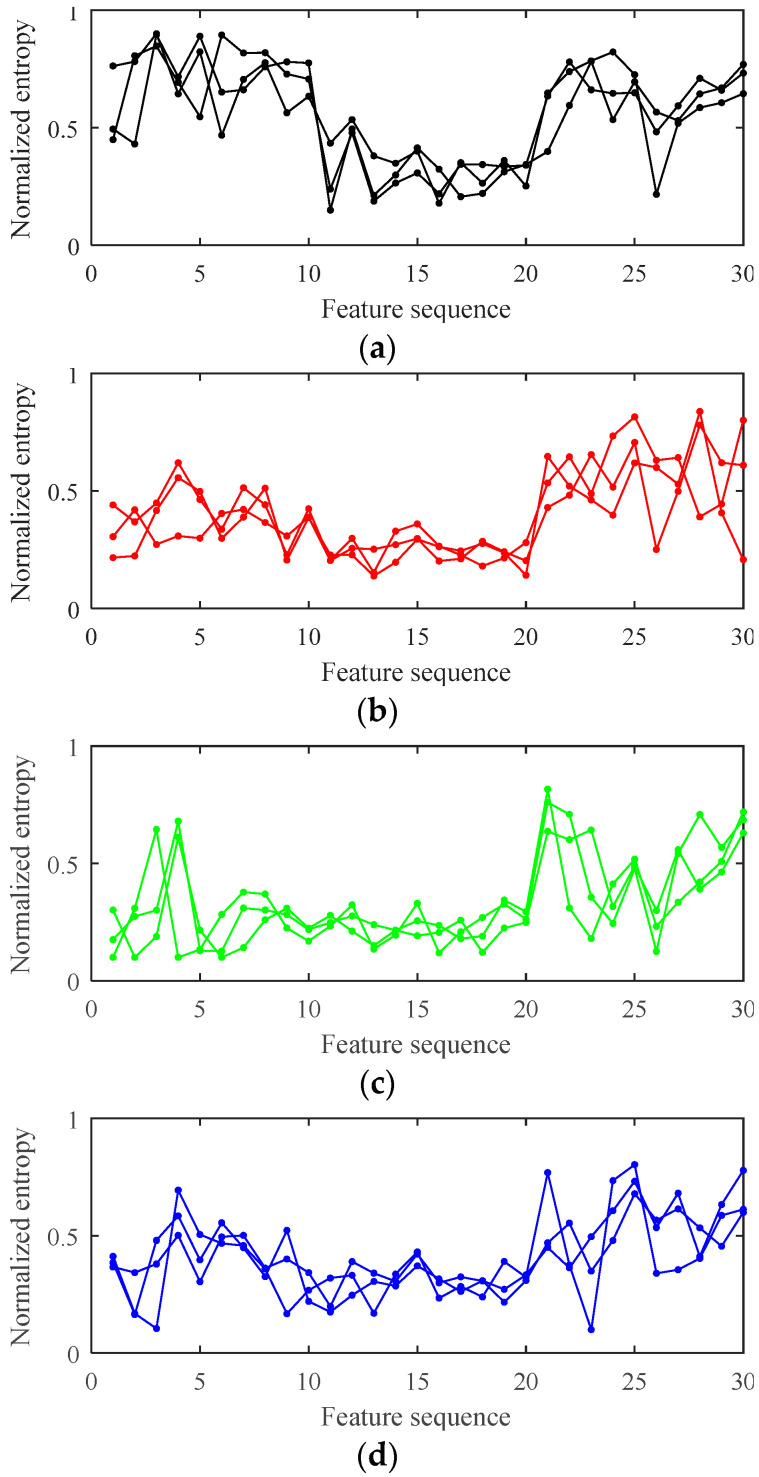
The normalized multi-feature entropy of the four types of vibration signals. (**a**) Normal state; (**b**) Fault I; (**c**) Fault II; (**d**) Fault III.

Due to the three kinds of Shannon entropies being in different dimensions, multi-feature entropy is normalized to show its distribution characteristics, as shown in Figure 8. For clarity, each type only displays three normalized multi-feature entropy vectors.

Figure 8 presents that the multi-feature entropy of different types of vibration signals has significant differences. However, it’s worthwhile to note that the distribution of features among the three faults still has some similarities. Since the three faults are all spring faults, they have similar fault mechanisms. Also, it can be seen from Figure 8 that the multi-feature entropy of different samples of the same type has a certain divergence. This phenomenon is because the HVCB has a complicated transmission system, so every experiment will be different.

#### 5.3.2. Multi-Feature Entropy Fusion

**Figure 9:**
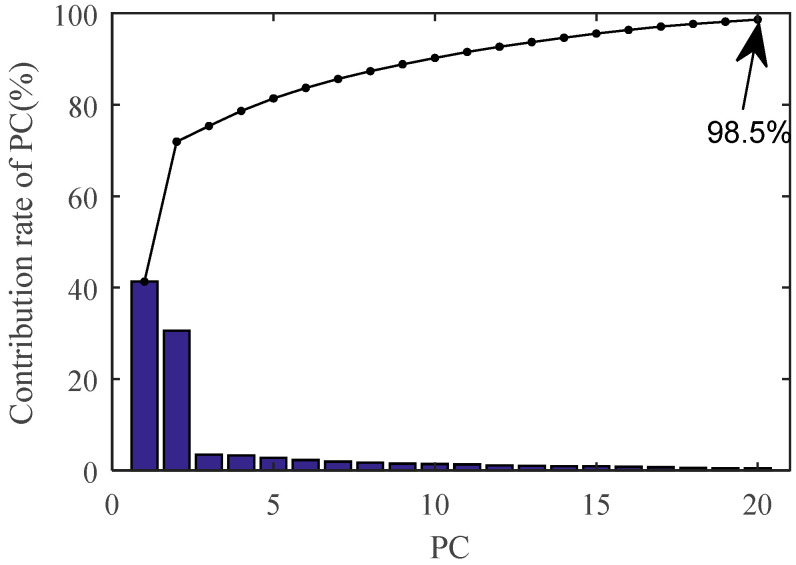
Cumulative contribution rate of principal component.

In this paper, the multi-feature entropy of the IMF is extracted from three different aspects. Although this method can avoid the problem of low accuracy and instability of single feature parameters, it may also causes the redundancy of features to a certain extent. Thus, PCA is used to reduce the dimension of feature vectors to achieve effective fusion of the multi-feature entropy. The cumulative contribution rate of the principal component (PC) is shown in Figure 9.

It can be seen that the cumulative contribution rate of the first twenty PCs has reached 98.5%, so the first twenty PCs are selected to form the feature vector.

**Figure 10:**
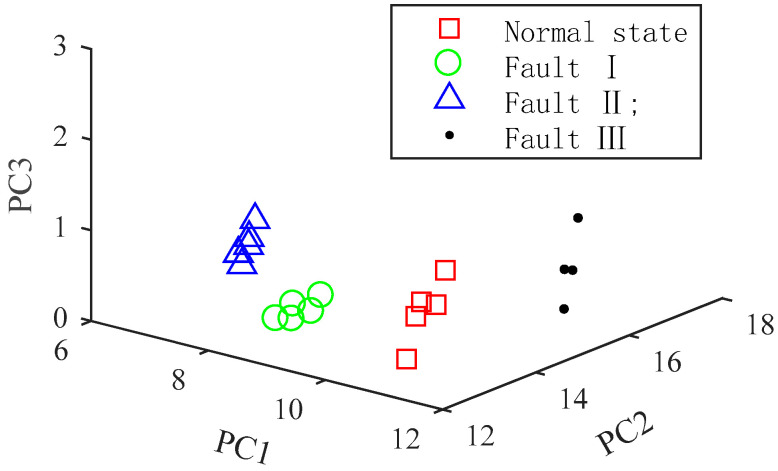
Spatial distribution of VMD-MFEF feature vectors.

The first three principal elements of the feature vectors are selected to reflect the spatial distribution of the feature vectors, as shown in Figure 10. For clarity, each type only displays five feature vectors. Figure 10 presents that different states are completely separated from each other in space, which indicates that the MFEF method is suitable. The distance between Faults I and II is the shortest, which indicates that the fault characteristics between them are similar.

**Figure 11:**
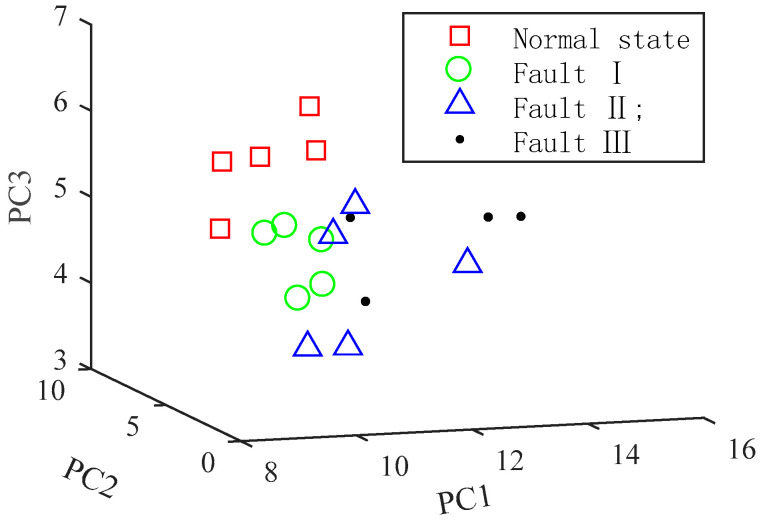
Spatial distribution of EMD-MFEF feature vectors.

In order to verify the effectiveness of VMD, EMD is used to decompose the vibration signal into 10 IMFs. Then, the MFEF method is used to extract the feature vectors of signals. The spatial distributions of EMD feature vectors are shown in Figure 11. It can be seen that aliasing exists in different states, so different states cannot be correctly distinguished.

### 5.4. Fault Classification Using the Hybrid Classifier

The feature vectors obtained in Section 5.3 are fed into the hybrid classifier for fault recognition and classification. The hybrid classifier consists of two classifiers: SVDD and SVM. In this paper, SVDD is employed to determine whether an unknown fault occurs in HVCBs, and SVM is employed to classify known states which include the normal state and known faults. SVDD and SVM should be trained first. For each type of vibration signal, 30 samples have been collected. In this case, 24 samples are selected as the training samples, and the other 6 samples are selected as the test samples.

#### 5.4.1. Unknown Fault Detection

In the actual operation environment, there may be some unrecorded unknown faults occurring in HVCBs. Once this happens, the classifier may recognize an unknown fault as a known state due to the lack of training samples of the unknown faults. Therefore, it is necessary to add a step to detect whether an unknown fault occurs in HVCBs before classifying samples. In this paper, SVDD is adopted to detect whether an unknown fault occurs in HVCBs.

To test the performance of SVDD, three cases are simulated: (a) Fault I is assumed to be the unknown fault; (b) Fault II is assumed to be the unknown fault; (c) Fault III is assumed to be the unknown fault. So the SVDD is trained by all training samples except the training samples of the assumed unknown fault. That means that testing samples have 18 samples of the known states and 6 samples of the unknown states in each case. The test results are shown in Table 1. The state determination accuracy is used to reflect the ability of SVDD to detect whether an unknown fault occurs in the HVCB.

From Table 1, it can be seen that the unknown detection accuracies of the three cases are 100%, 87.5%, and 100% respectively. The reason for the low detection accuracy of the second case can be explained by Figure 9. From Figure 9, it can be seen that the distance between Faults I and II is the shortest in the feature space, which indicates that the fault characteristics between Faults I and II are similar. And the result of the second case will be further analyzed in Section 5.4.2.

In order to show the superiority of SVDD, a comparison is made between SVDD and SVM. The detection results are shown in Table 2 when Fault III is assumed to be the unknown fault.

Table 2 shows that SVM has no ability to detect the new fault, while SVDD can completely recognize it. One-class support vector machine (OCSVM) is also a one-class classifier, which also has many applications in fault diagnosis [16]. Another comparison is made between SVDD and OCSVM. The diagnosis results are shown in Table 2.

As illustrated in Table 2, the detection accuracy of SVDD is significantly higher than that of OCSVM. This indicates that SVDD has a distinct advantage in the detection of unknown faults of HVCBs.

#### 5.4.2. Known States Recognition and Classification

If the sample is recognized as an unknown fault by SVDD, the sample will be imported into the area of known states; otherwise, it will be imported into the area of unknown states; then, samples of the area of known states will be classified by SVM. 

Table 3 shows the classification results of the test samples by SVM It can be seen that all test samples are classified correctly. Thus, SVM has a good ability to identify the state of HVCBs, and the feature extraction method proposed in this paper is suitable.

The performance of the MFEF feature vector and the single feature vector is compared. Since the performance of energy entropy is better than others, it is selected as the single feature vector. The results of the experiment are shown in Table 3. According to the results in Table 3, the classification accuracies (CA) of the three-feature extraction method are all 100%, which means that the normal state is easy to distinguish. The average classification accuracies (ACA) of using both the single feature vectors and the multi-feature vectors are lower than that of using the multi-feature fusion vectors. This is because the MFEF method proposed in this paper can reflect the characteristics of vibration signals from different aspects, and can avoid the redundancy of features.

Similarly, a contrast comparison is made between VMD and EMD. After applying 10-layer EMD to original vibration signals, the EMD-MFEF vectors are calculated. The comparison results are shown in Table 4. Based on Table 4, it can be seen that the classification accuracy of VMD-MFEF is superior to that of EMD-MFEF.

In order to further analyze the results of the case that Fault II is assumed to be the unknown fault in Section 5.4.1, the SVM is trained by all training samples except the training samples of Fault II. The feature vectors in the known states area that were obtained in Section 5.4.1 are fed into SVM. The known states area includes 6 samples of the normal state, 6 samples of Fault I, 3 samples of Fault II, and 6 samples of Fault III. The classification result shows that the samples of Fault II are all recognized as Fault I, which is shown in Table 5. Although Fault II is not completely detected by SVDD in Section 5.4.1, the classification result of Table 5 is acceptable, since the question of whether the HVCB is healthy or not is addressed correctly.

## 6. Conclusions

Previous studies have documented the effectiveness of vibration signals in fault diagnosis of HVCBs. However, most of these studies only extracted the single feature as feature vectors, and paid little attention to the detection of unknown faults. This paper proposes a novel fault diagnosis method of HVCBs based on VMD-MFEF and hybrid classifier. The experiments results demonstrate the MFEF method can extract fault information precisely, and that the hybrid classifier constructed with SVDD and SVM can not only accurately classify the fault types, but also detect whether unknown faults occur in HVCBs. Conclusions can be drawn as follows:

(1) The fault signatures can be extracted precisely by using the VMD-MFEF method. Compared with the EMD-MFEF feature vectors, the VMD-MFEF feature vectors have better spatial distribution in the feature space. Different states are completely separated from each other in feature space.
(2) To test the stability of SVDD, the three faults simulated in this paper are assumed to be unknown faults. The detection accuracies of the unknown fault in the three cases are 100%, 87.5%, and 100% respectively. The reason for the low detection accuracy in the second case is that the spatial positions of Faults I and II are close in the feature space, which indicates that the fault characteristics between them are similar. Although the two faults can be correctly classified in the classification of known states when both of them are involved in the training of SVM, the maintenance personnel should pay attention to the similarity between the two faults’ characteristics to avoid the occurrence of error diagnosis. Compared with SVM and OCSVM, SVDD has a distinct advantage in the detection of unknown faults of HVCBs.
(3) Compared with the single feature extraction method, the proposed MFEF method is superior in terms of feature extraction. The experimental results of the classification of known states show that the faults classification accuracy of the MFEF method achieves an accuracy of 100%, while the single-feature method only achieved an accuracy of 75%.

In the actual operating environment, it is easy to collect many vibration signals of the normal state, but the vibration signals of fault states are difficult to collect. So, there is a problem of a sample imbalance. In future, we will study the problem of fault identification and classification under the condition of a sample imbalance. Additionally, compound faults of HVCBs will also be an important and interesting future research subject.

## Figures and Tables

**Table 1:** Diagnosis results of the unknown fault using SVDD.

Cases	Known States	Unknown States	Accuracy
a	18	6	100%
b	21	3	87.5%
c	18	6	100%

**Table 2:** Diagnosis results of the unknown fault using different classifiers.

Classifier	Known States	Unknown States	Accuracy
SVDD	18	6	100%
SVM	24	0	0
OCSVM	14	10	83.33%

**Table 3:** Classification accuracy of different feature method.

Fault States	Single Feature		Multi-Feature		MFEF	
	CA	ACA	CA	ACA	CA	ACA
Normal state	100%	75%	100%	95.83%	100%	100%
Fault I	50%		83.33%		100%	
Fault II	83.33%		100%		100%	
Fault III	66.67%		100%		100%	

**Table 4:** Classification accuracy of different signal processing method.

Fault States	VMD-MFEF		EMD-MFEF	
	CA	ACA	CA	ACA
Normal state	100%	100%	83.33%	79.17%
Fault I	100%		83.33%	
Fault II	100%		66.67%	
Fault III	100%		83.33%	

**Table 5:** Classification accuracy of known state area.

Classifier	Normal State	Fault I	Fault III
SVM	6	9	6
